# A Two-Years' Survey on the Prevalence of Tuberculosis Caused by *Mycobacterium caprae* in Red Deer (*Cervus elaphus*) in the Tyrol, Austria

**DOI:** 10.5402/2012/245138

**Published:** 2012-10-22

**Authors:** Karl Schoepf, Wolfgang M. Prodinger, Walter Glawischnig, Erwin Hofer, Sandra Revilla-Fernandez, Johannes Hofrichter, Johannes Fritz, Josef Köfer, Friedrich Schmoll

**Affiliations:** ^1^Austrian Agency for Health and Food Safety (AGES), Institute for Veterinary Disease Control Innsbruck, Technikerstrasse 70, 6020 Innsbruck, Austria; ^2^Division of Hygiene and Medical Microbiology, Medical University of Innsbruck, Fritz-Pregl-Stra*β*e 3/3, 6020 Innsbruck, Austria; ^3^Austrian Agency for Health and Food Safety (AGES), Institute for Veterinary Disease Control Mödling, Robert Koch Gasse 17, 2340 Mödling, Austria; ^4^Austrian Agency for Health and Food Safety (AGES), Data, Statistics and Risk Assessment, 8020 Graz, Austria; ^5^Veterinary Department, Regional Government of the Tyrol, 6600 Reutte, Austria; ^6^Institute for Veterinary Public Health, University of Veterinary Medicine, Veterinärplatz, 1210 Vienna, Austria

## Abstract

A survey of 143 hunter-harvested red deer for tuberculosis was conducted in an Alpine area in Western Austria over two subsequent years. There, single tuberculosis cases caused by *Mycobacterium caprae* had been detected in cattle and red deer over the preceding decade. The area under investigation covered approximately 500 km^2^, divided into five different hunting plots. Lymph nodes of red deer were examined grossly and microscopically for typical tuberculosis-like lesions and additionally by microbiological culturing. Executing a detailed hunting plan, nine *M. caprae* isolates were obtained. Six out of nine originated from one single hunting plot with the highest estimated prevalence of tuberculosis, that is, 23.1%. All isolates were genotyped by mycobacterial interspersed repetitive unit—variable number of tandem repeat (MIRU-VNTR) typing of 24 standard loci plus VNTR 1982. All nine isolates belonged to a single cluster termed “Lechtal” which had been found in cattle and red deer in the region, demonstrating a remarkable dominance and stability over ten years. This is the first report on a systematic prospective study investigating the prevalence and strain variability of *M. caprae* infection in red deer in Austria and in the Alpine countries.

## 1. Introduction

Tuberculosis (TB) is a chronic infectious disease in wildlife caused by bacteria of the *Mycobacterium tuberculosis* complex (MTC). Several members of the MTC can be distinguished, *Mycobacterium caprae* being one of them. *M. caprae, *the aetiological agent found in our study, can infect a wide range of domestic animals, wild animals, and humans [1–3]. Infection in humans with *M. caprae* appears to be relatively rare and occur preferentially in older patients [4]. Only very few documented autochthonous human cases have been found outside of continental Europe [1]. The body of literature on *M. caprae *is relatively small, as *M. caprae* was described as a new subspecies in the MTC only recently in 2003 [3].

Similarly, the role of wildlife in the maintenance and spread of TB is far better studied for *M. bovis,* [5–9], although most of the findings probably hold true for *M. caprae* and are therefore briefly highlighted in the following. The best-known European examples of TB reservoir species in wildlife are the Eurasian badger (*Meles meles*) in the UK and the wild boar (*Sus scrofa*) in Spain [10, 11]. In 1964, Bischofberger and Nabholz [12] already reported cases of tuberculosis in roe deer (*Capreolus capreolus*) from Switzerland that appeared to have caused infections in cattle. A recent farm and slaughter survey of captive deer in Switzerland showed, however, no evidence of TB infection [13]. TB has also been reported more recently in roe deer from Spain, Austria, and Italy, respectively [7, 14, 15], and in red deer and wild boar from France (Normandy) [8]. Nonruminant hosts such as the brush tailed possums (*Trichosurus vulpecula*) in New Zealand and the Eurasian badger in Great Britain and Ireland are undoubtedly the primary wildlife reservoir hosts of TB (exclusively *M. bovis*, not *M. caprae*) [16–18]. A self-sustaining reservoir of TB in wild, free ranging white tailed deer (*Odocoileus virginianus*) was identified in Michigan, USA [19–21]. TB has been under observation in South Africa's Krüger National Park, primarily in buffalo, but spreading to lions and other valuable wildlife species putting the entire lion population at risk [22]. In most cases, however, deer are thought to be spill-over end hosts [5, 23, 24]. 

TB in wildlife is of particular importance in countries where eradication programmes have eliminated this zoonosis in cattle. Eradication of TB in livestock has been impeded in several countries by the presence of TB infection in wild species [25, 26]. The same experience was observed in the area under surveillance described in this paper. Austria has been officially declared free from bovine tuberculosis in 1999 due to a comprehensive test and slaughter programme in cattle which started in the 1950s. Freedom from infection is currently being monitored through meat inspection and abattoir surveillance. Until 1999, the Austrian Agency for Health and Food Safety (AGES) had observed only single cases of tuberculosis in red deer within the provinces of the Tyrol and Vorarlberg, respectively. Due to missing surveillance in wildlife, TB at a low prevalence went undetected for a long period. Since the year 2000, individual cattle originating from the Tyrolean District of Reutte, slaughtered, and meat-inspected in the neighbouring regions in Germany, has shown gross lesions due to TB. The same infectious agent as in red deer could be detected. Because of these preliminary observations, it was evident that an ongoing epidemic of *M. caprae* infection in free ranging red deer was present [27]. In 2008, an increase in TB cases in domestic cattle caused by *M. caprae* has been recorded on several farms within the District of Reutte in the northwest of the Tyrol, particularly in and around the village of Steeg. The presence of this reemerging disease in cattle was the trigger to initiate a coordinated long-term cross-sectional study in red deer. Together with all relevant stakeholders, systematic sampling took place in the hunting seasons between 2008 and 2009. Parallel to this initiative, an official TB control campaign in cattle using tuberculin skin testing (TST) was launched by the Tyrolean regional veterinary authorities in 2009. All cattle holdings in the four western districts of the Tyrol (Reutte, Landeck, Imst, and Innsbruck Land) which were declared surveillance zones by the Austrian Ministry of Health were included. This control programme is still on-going.

The main goal of this targeted surveillance was to collect conclusive data on the current prevalence of TB infection in red deer population in the northwestern part of the Tyrol, applying standardised sampling and necropsy techniques and uniform diagnostic methodology in microbiology and molecular typing. This report presents the first data on the prevalence of *M. caprae* in red deer population in the northwestern part of the province of Tyrol. An outlook for the future strategy on combatting TB infection in wildlife is given.

## 2. Materials and Methods

The survey was initiated in August 2008 in cooperation with professional hunters, local veterinarians, and staff from public veterinary authorities. A network of local hunters cooperating with local veterinarians was established throughout the observation area. Red deer of specific age and sex were trophy hunted and cropped according to the current hunting plan issued every year by the local district authorities. Lymph nodes from red deer carcasses were submitted by veterinarians for laboratory investigations to the AGES. 

### 2.1. Study Area and Sampling Plan

**Figure 1:**
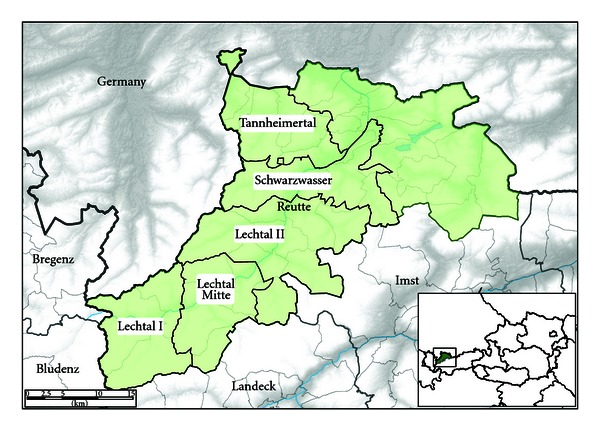
Study area and hunting plots. Five named hunting plots were defined within the District of Reutte (green area) in the province of the Tyrol, Austria. The study area has a border to Germany (Bavaria) in the north, to the Austrian province of Vorarlberg in the west (districts of Bregenz and Bludenz, respectively), and to other Tyrolean districts in the south.

A risk-based sampling plan was issued by the AGES department data statistics and risk assessment covering an observation period of two years. The District of Reutte covers an area of total 1,236.82 km². The area under investigation in the District of Reutte of approximately 500 square kilometres was divided in five different hunting plots: Lechtal I, Lechtal Mitte, Lechtal II, Tannheimertal, and Schwarzwasser (see Figure 1). Each included smaller game properties. The sampling plan was followed in the hunting seasons 2008 and 2009 (Table 1).

Estimated population size can be evaluated either by animal count at the feeding sites or by hunting bag data. The total red deer population in the District of Reutte counts approximately 6.900 animals. Especially in the western part of the District of Reutte the red deer population has increased dramatically due to the fact that surplus animals have not been harvested neglecting the guidelines issued by the district authorities. Estimated population density of red deer in the District of Reutte based on hunting bag data revealed 5.6 deer per 100 hectare (Chris Walzer, Vienna, personal communication). In the present survey, 143 hunted animals of different age, sex and from different geographical locations, showing no signs of apparent disease were included. From each collected carcass sex, age, date of collection and the location of hunting (global positioning system (GPS) data) were recorded. The carcasses were classified into following age and sex groups, according to the Tyrolean hunting law: stag aged >9 years, stag aged 5–9 years, stag aged <5 years, hind <2 years, hind >2 years, and calves <1 years. In calves no sex differentiation was recorded.

### 2.2. Pathology and Histopathology

Hunted red deer first underwent a systematic examination by the local veterinarian, and then organ material was presented to the laboratory. Retropharyngeal, tracheobronchial, mediastinal, mesenterial, and ileocecal lymph nodes were identified and submitted refrigerated to the AGES laboratory. Samples for histology were fixed in 6% buffered formaldehyde and embedded in paraffin. 5 *μ*m thick slices were stained with haematoxylin/eosin and with Ziehl-Neelsen stain for acid-fast bacilli, respectively, according to standard methodology.

### 2.3. Microbiology and Culture Identification Methods

Refrigerated portions of different lymph nodes were submitted to the Austrian National Reference Laboratory for Bovine Tuberculosis (NRL) for mycobacterial culture. Routine techniques were applied as earlier described [27] to isolate mycobacteria from tissue samples. MTC species differentiation was performed with the GenoType MTBC test system (Hain Lifescience GmbH, Nehren, Germany) [28] following the manufacters' instructions. 

### 2.4. Molecular Typing

*M. caprae *isolates were genotyped by two methods: first by spoligotyping [29] and secondly by mycobacterial interspersed repetitive unit—variable number of tandem repeat typing (MIRU-VNTR typing)—a high discriminative method to determine the copy numbers in 24 variable number tandem repeat (VNTR) loci [30, 31]. In addition, a 25th locus (i.e., VNTR 1982) was determined with the primers described by van Deutekom et al. [32]. MIRU-VNTR typing was carried out as single PCR for each locus and analysed by gel electrophoresis, as described earlier [1]. For the digital comparison of fingerprints, Bionumerics software version 3.5 (Applied Maths, St. Martens Latem, Belgium) was used. Spoligotyping results were checked with the UK *M. bovis *database [33] for matching patterns.

## 3. Results

### 3.1. Culture Positive Animals and Prevalence

*M. caprae *was successfully cultivated from 9 of the 143 analysed red deer. The prevalence of TB infection in the hunting plot Lechtal I was 23.1% and in Lechtal Mitte 9.7%, respectively. The hunting plots Lechtal II, Tannheimertal, and Schwarzwasser yielded no TB culture positive animal (Table 1). Three positive animals could be detected in the male group aged 5–9 year and three animals in females older than 2 years. Two positive animals were found in males younger than 5 years and one in female younger than 2 years.

### 3.2. Pathology and Histopathology

Gross pathology and histology results for all 143 animals were recorded. Purulent abscesses which varied remarkably in size were detected in retropharyngeal lymph nodes from seven culture positive animals. In one animal purulent abscesses could also be found in the mediastinal lymph nodes. Two additional culture positive animals showed no characteristic lesions. Microscopically, lymph node lesions showed mostly thin-walled abscesses containing pus with singular calcification and giant and epithelioid cells in the capsule. All histopathology positive tissue samples were subjected to Ziehl-Neelsen staining, but no acid-fast bacteria could be detected.

### 3.3. Molecular Typing

**Figure 2:**
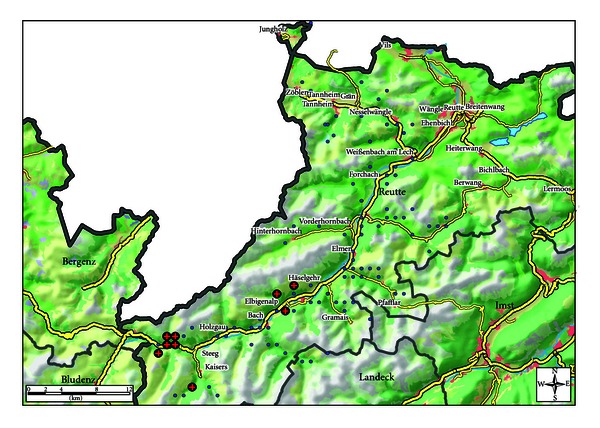
Geographical distribution of culture positive (red cross on black bullet) and culture negative (blue bullet) animals investigated in the study area in the District of Reutte, province of the Tyrol, Austria. Culture positive animals were confined to the hunting plots “Lechtal I” and “Lechtal Mitte.”

MTC differentiation by the GenoType MTBC test system revealed all nine isolates to be *M. caprae*. All nine showed the same spoligotype pattern SB0418 according to the UK *M. bovis* database [33], a pattern typical for *M. caprae *and widespread in Europe (and thus noninformative). All isolates furthermore belonged to one dominant MIRU-VNTR pattern termed “Lechtal” upon determination of copy numbers for 24 loci. Six out of nine were identical in all 24 loci, and three further ones were a minimal variant thereof (i.e., identical with the consensus type “Lechtal” at 23 out of 24 loci). Even addition of a 25th VNTR 1982 to the 24-loci standard panel did not improve strain discrimination as all nine isolates were identical in this locus. The consensus type “Lechtal” and its variants are shown in Table 2. Only one of the variant patterns had been observed earlier: this pattern “Kaisers” had been seen in two deer in the Kaisers side valley south of Steeg (see Figure 2) in 2006. The matching animal isolate from this study originated from a spot geographically close to Kaisers. 

## 4. Discussion

TB in wildlife is a common problem in several European Union Member states constituting a continuous source for reinfection of cattle. It is being considered as an emerging disease of major economic and public health importance. Beginning in 1999 single cases of TB due to *M. caprae* in red deer within the provinces of the Tyrol and Vorarlberg, respectively, were observed [2, 27]. A serious outbreak of TB in singular cattle holding was observed at the beginning of 2008 in the village of Steeg (District of Reutte, Tyrol). This case had a substantial impact on veterinary public health and on animal trade. Since then a compulsory test and slaughter programme within a defined geographical area were reintroduced. Several farms from which animals had contact to the originally TB infected holding further TST-positive animals were detected. 

Our findings show that the prevalence rate in red deer in the study area varies depending on the respective hunting plot area from 23.1% to 0%. As the data indicate, the western part of the study area appears to be a hot spot (Figure 2). All nine *M. caprae *isolates from this study belonged—allowing for a minimal divergence in 1 of 25 loci—to one genotype (Lechtal) which has been identified in the area since 1999 [1, 27]. In the same time period, no other genotype was detected in regional animal *M. caprae *isolates. One of the three minimal variants (pattern “Kaisers”) has been observed earlier and may reflect the establishment of a mini-subcluster in the side-valley of Kaisers. Altogether, type “Lechtal” shows a remarkable genetic stability over more than a decade and to cause at least the vast majority of regional deer TB cases. Furthermore, the “Lechtal” genotype has not been found in a comprehensive study on *M. caprae *genotypes in Europe except in Austria (the Tyrol and Vorarlberg, resp.), bordering regions of Bavaria and once in northern Italy [1].

Among samples from red deer in Bavaria (Germany) that have been studied in a recently approved PhD thesis, a tuberculosis prevalence of 0.91% was identified: in that study all samples from cattle and red deer that tested positive for *M. caprae* originated from the region close to the border with the Austrian District of Reutte [34]. *M. caprae* may infect multiple species including several nonruminant species, such as the red fox (*Vulpes vulpes*) and wild boars (*Sus scrofa*) [16]. The latter are not common in the province of the Tyrol, as their habitat is restricted to eastern Austria. In Spain, *M. caprae* infection in wildlife is well documented [14]. In this country *M. caprae* is also common in domestic animals, mainly in goats, but also in cattle and pigs. Also in Croatia, *M. caprae *has been detected in cattle and pigs [35].

Due to our observations and due to the identical genotypes found in *M. caprae* isolated from red deer as well as from cattle, we postulate that domestic cattle became infected over the years through contact with free ranging red deer which have reached the status of maintenance hosts. Infection in deer may persist by intraspecies transmission and is the source of infection for other species [22, 25]. When assessing the risk of transmitting TB from infected wildlife to cattle, interaction of wildlife with cattle within the same ecosystem is crucial. The management practice of annual transhumance in alpine regions, which affects over 50% of the entire cattle population of the Tyrol, plays an important role in facilitating the transmission, either by direct or indirect contact between wildlife and cattle. Alpine common pastures are being grazed on by cattle together with red deer during summer period. Cattle may become infected through contamination of the environment close to feeding sites by excretions of infected wildlife such as faeces, urine, pus, or sputum. *M. caprae* infection can be contracted mainly by two routes: aerosol inhalation and ingestion. Reviewing *M. bovis* transmission from and to wild animals, Corner summarizes the transmission process between deer to cattle as unclear: although he estimates the risk of aerosol transmission to cattle to be more pervasive than the risk of infection by ingestion, aerosol transmission would require close interaction of cattle and deer in both time and space, and moreover cattle are relatively insensitive to oral challenge [23]. Several alpine pastures where red deer and cattle originating from different farms graze together are located in the specified hunting areas covered by the study. In particular wildlife winter feeding sites, where contaminated food and salt licks are available, might constitute another potential source of infection [36]. Prolonged crowding of red deer around feeding sites provides an advantageous opportunity for deer to deer contact and enhances intraspecies transmission. In the situation found in our study, the most important factors include population size, supplementary feeding during winter, and the rutting season. Winter feeding is practiced to prevent migration and decrease the death rates during winter, in attempt to keep deer population high. According to cropping numbers red deer population in the western part of the District of Reutte has steadily increased over the years to an unacceptably high level. 

Management of wildlife disease can be classified into four basic categories: prevention, control, eradication, and doing nothing (laissez faire) [25, 37]. The issue of TB in wildlife cuts across a variety of stakeholder interests. Within an atmosphere of conflict and uncertainty, wildlife disease reservoirs for *M. caprae* often pose a “wicked problem” [37]. This obstacle will be experienced by all stakeholders, when trying to implement control strategies. The greatest risk at present is that infected red deer will spread to other geographical locations when targeted culling and hunting activities are being intensified. Outside of the province of the Tyrol, *M. caprae* infection in red deer was recorded to a lesser extent in the Austrian province of Vorarlberg, and also in the neighbouring German Federal State of Bavaria [1, 34, 38]. Thus, the threat of TB to alpine wildlife is currently under investigation in a common European ERANET project involving parts of Austria, Germany, Italy, and Switzerland (http://tb-alpine-wildlife.org/). 

The outcome of this survey provides first data on the prevalence of *M. caprae* in a well-defined geographical area. Further studies are necessary to understand the epidemiology and the distribution pattern of *M. caprae *infection in red deer. 

## Figures and Tables

**Table 1:** Numbers of sampled animals per each hunting plot in the study area. The number of culture positive animals among each sampled population is given in brackets.

	Male			Female		Calves	Total
Age category in years	<5 (III)	5–9 (II)	>9 (I)	<2 (III)	>2 (II)	<1	
Plot “Lechtal I”	8 (2)	2 (2)	1	2	8 (2)	5	26 (6)
Plot “Lechtal Mitte”	6	5 (1)	3	2 (1)	7 (1)	8	31 (3)
Plot “Lechtal II”	9	9	3	4	9	10	44
Plot “Tannheimertal”	5	6	2	2	7	4	26
Plot “Schwarzwasser”	2	3	2	1	5	3	16

Total study area	30 (2)	25 (3)	11	11 (1)	36 (3)	30	143 (9)

**Table 2:** MIRU-VNTR consensus type “Lechtal” given as copy numbers for the standard 24 loci. Minimal variations in single loci occurring in 3 of the 9 isolates are shown as variants.

VNTR locus name	Copy number	Variants
MIRU 2	2	
MIRU 4	2	
MIRU 10	6	5
MIRU 16	4	
MIRU 20	2	
MIRU 23	4	
MIRU 24	2	
MIRU 26	4	
MIRU 27	3	
MIRU 31	3	
MIRU 39	2	
MIRU 40	2	
VNTR 424	4	2
VNTR 577	5	
VNTR 1982^a^	3	
VNTR 2401	4	
VNTR 3690	1	
VNTR 4156	3	
VNTR 1955	2	
VNTR 2163^b^	5	4^b^
VNTR 2165	5	
VNTR 2347	3	
VNTR 2461	3	
VNTR 3171	2	
VNTR 4052	3	

^a^VNTR 1982 (not included in the standard loci panel) was determined additionally.

^b^ Denotes pattern “Kaisers” (see text).
